# Assessment of transition readiness in adolescents and young adults with chronic health conditions

**DOI:** 10.1186/s12969-017-0197-6

**Published:** 2017-09-09

**Authors:** Paul T. Jensen, Gabrielle V. Paul, Stephanie LaCount, Juan Peng, Charles H. Spencer, Gloria C. Higgins, Brendan Boyle, Manmohan Kamboj, Christopher Smallwood, Stacy P. Ardoin

**Affiliations:** 1 Division of Rheumatology, Nationwide Children’s Hospital, 700 Children’s Drive, Columbus, OH 43205 USA; 2 The Ohio State University College of Medicine, 370 W. 9th Avenue, Columbus, OH 43210 USA; 3 The Ohio State University Center for Biostatistics, 320 Lincoln Tower, Columbus, OH 43210 USA

**Keywords:** Adolescent, Young adult, Adult, Transition readiness, Chronic health conditions

## Abstract

**Background:**

Transition from pediatric to adult health care is a vulnerable period for adolescents and young adults. Challenges include paucity of validated measures to assess patients’ transition readiness. We evaluated the Transition Readiness Assessment Questionnaire (TRAQ) in adolescents and young adults with rheumatic, gastrointestinal, and endocrine disorders. We examined whether baseline TRAQ scores and other demographic variables predicted transition to adult care over a three year follow up period.

**Methods:**

In this descriptive study at a single institution, eighty-nine adolescents at a single pediatric academic medical center completed demographic and medical history surveys and the TRAQ and were followed over 3 years by telephone interview to determine whether they had transitioned to adult subspecialty care. Transition was defined as attending at least one adult subspecialty appointment. Multivariable logistic regression and Cox proportional hazards regression models were used to determine whether TRAQ scores predicted time to transition.

**Results:**

Of the participants, 56% had rheumatic, 21% endocrine, and 23% gastrointestinal conditions. The TRAQ self-management domain score was not significantly associated with age, gender, socioeconomic status, or specialty. The TRAQ self-advocacy score increased with age. Baseline TRAQ scores did not predict transition or time to transition over three years.

**Conclusion:**

In this cohort of adolescents and young adults who were 16 to 23 years of age at enrollment, 48% transitioned to adult care over three years of follow up. Nearly half reported not discussing transition with provider or seeing provider independently for part of visit. Older age but not other demographic variables nor baseline TRAQ score predicted transition or time to transition to an adult subspecialty provider; however, a there was a trend towards shorter time to transition with the highest quartile TRAQ scores.

## Background

In the United States, approximately 500,000 adolescents with special health care needs reach the age of 18 each year and ultimately need to transition from pediatric to adult health care [1]. The transition from pediatric care to adult care is often a challenging time for patients with chronic medical conditions and their providers [2]. Many adolescents and young adults lack important self-management and health care utilization skills [3–5] and are insufficiently prepared to move to adult care [6, 7].Gaps in health care can lead to increased morbidity and mortality [8–14].

A transition process which starts in early adolescence can help patients develop self-care, self-advocacy, and decision-making skills, and the need for such a process has been acknowledged [15]. However, despite several efforts to improve the transition process, gaps remain. The 2001 National Survey of Children with Special Health Care Needs (NS-CSHCN) found that only 15% of youth reported that their doctor had discussed changing needs in adulthood, including the need to transition to an adult doctor [16]. A 2006 follow up NS-CSHCN survey identified that only 40% of youth reported meeting the medical transition outcome measure (a composite of whether the provider had discussed transition to adult care, health care needs, health insurance, and had encouraged the patient to take responsibility for his/her own care) [1, 6]. A 2014 systematic review concluded that most published studies aiming to improve transition process failed to address the “triple aim” of impact on health outcomes, patient satisfaction, and cost [17]. The Agency for Health Care Research and Quality (AHRQ) has acknowledged that identification of optimal transition processes and interventions are limited by the lack of a robust evidence base [18].

In 2002, the American Academy of Pediatrics (AAP), American Association of Family Physicians (AAFP) and American College of Physicians-American Society of Internal Medicine (ACP) jointly recommended that providers regularly assess transition readiness skills using an objective measure [15]. Recently, new tools have been developed to measure patients’ readiness to transition to adult care [19–21]. The Transition Readiness Assessment Questionnaire (TRAQ), developed by Sawicki and colleagues, is one of these tools.

The TRAQ was initially studied at the Jacksonville Health and Transition Services Clinic at the University of Florida in Jacksonville in a cohort of patients with several pediatric-onset chronic health conditions including spina bifida, cerebral palsy, diabetes mellitus, cystic fibrosis, sickle cell disease, seizure disorders, and autism as well as in patients attending the Cystic Fibrosis Center at Boston Children’s Hospital. In the initial study, TRAQ self-management (Domain 1) and self-advocacy (Domain 2) scores were positively associated with age and the TRAQ self-advocacy score was higher in females [22]. Patient education interventions can improve TRAQ scores as shown in studies of patients with heart disease and cystic fibrosis [23, 24]. A revised 20-item TRAQ questionnaire (TRAQ 5.0) found no significant association between TRAQ scores and race, ethnicity, or insurance types [3]. The TRAQ is one of the few available validated, patient-reported, disease non-specific transition readiness tools [25].

In this study, we aimed to evaluate whether demographic variables or baseline TRAQ scores predicted transition or time to transition over 3 years in a cohort of adolescents and young adults with rheumatic, endocrine, and gastrointestinal (GI) disorders.

## Methods

### Patient population

Institutional Review Board approval was obtained for this study, and informed consent was obtained for all participants. Consecutive patients with rheumatologic, endocrine and GI chronic diseases expected to require adult subspecialty care were included and followed for three years from 2011 to 2015. All participants were recruited from outpatient pediatric specialty clinics at standard of care clinic visits. At the time of the study, none of the involved clinics had a formal transition policy or process. Participants had to be English speaking, aged 16 to 25 years, and able to complete the TRAQ instrument.

### Study design

This is a descriptive, longitudinal study performed at a single academic institution. At the baseline visit, participants completed demographic and medical history questionnaires. Medical history was confirmed by chart review. Participants then completed the TRAQ version 4.1. Providers were blinded to TRAQ scores. After the baseline visit, study personnel contacted participants every 6 months over a three year period by telephone interview. Study personnel inquired whether participants had been seen by an adult subspecialty provider. Attendance at the adult subspecialty provider visit was confirmed by chart review or having the adult providers document study participant clinic attendance.

### TRAQ instrument

The TRAQ 4.1 is a 29-item patient-reported measure of transition readiness across two domains: self-management (Domain 1) and self-advocacy (Domain 2) [22]. Items included in the TRAQ 4.1 are listed in Table 1. Answers are reported on a 1 to 5 scale based on the Stages of Change Model ranging from “I do not need to do this” to “I always do this when I need to.” A sixth option of “Not needed for my care” was also allowed for questions not applicable to an individual patient. Within each domain, the responses to each item were averaged to produce a domain score ranging from 1 to 5. The TRAQ is not specific to any diagnosis or specialty [22].

**Table 1:** Items Included in the Transition Readiness Assessment Questionnaire, version 4.1^a^

Domain 1: Skills for Chronic Condition Self-Management	Domain 2: Skills for Self-Advocacy and Health Care Utilization
Do you fill a prescription if you need to?	Do you fill out the medical history form, including a list of your allergies?
Do you know what to do if you are having a bad reaction to your medications?	Do you keep a calendar or list of medical and other appointments?
Do you pay or arrange payments for your medications?	Do you tell the doctor or nurse what you are feeling?
Do you take medications correctly and on your own?	Do you ask questions of the doctor, nurse or clinic staff?
Do you reorder medications before they run out?	Do you make a list of questions before the doctor’s visit?
Do you call the suppliers when there is a problem with the equipment?	Do you request the accommodations & support you need at school or work?
Do you order medical equipment before they run out?	Do you apply for a job or work or vocational services?
Do you arrange payment for the medical equipment and supplies?	Do you get financial help with school or work?
Do you call the doctor’s office to make an appointment?	Do you help plan or prepare meals/food?
Do you call the doctor about unusual changes in your health?	Do you help keep home/room clean or clean up after meals?
Do you apply for health insurance if you lose your current coverage?	Do you use neighborhood stores and services?
Do you know what your health insurance covers?	Do you use community support services or advocacy services when you need them?
Do you manage your money and budget household expenses?	
Do you reorder medications before they run out?	

^a^ Respondents select one of the following answers for each item: “Not needed for my care,” “No, I do not know how,” “No, I do not know how but I want to learn,” “No, but I am learning to do this,” “Yes, I have started doing this,” or “Yes, I always do this when I need to”

### Statistical analysis

Baseline demographic and transition-related characteristics were compared across groups by using t-tests and chi-square tests as appropriate. Univariate logistic regression models were used to test the effect of potential predictors on transition. Odds ratios describing the odds of transition and 95% confidence intervals were estimated from these univariate logistic regression models. Variables with *p*-value <0.15 in the univariate logistic regression model were entered into multivariate models. Cox proportional hazards regression models were used to examine the effect of TRAQ Domain 1 score and TRAQ Domain 2 score on the “hazard” function of transition after entering the study, while controlling for patient’s age at enrollment. A two-sided significance level of α = 0.05 was used for all tests. Analyses were performed by using SAS version 9.3 (SAS Institute, Cary, NC).

## Results

### Baseline characteristics

As outlined in Table 2, the 89 participants were 65% female, 81% Caucasian, 97% non-Hispanic. The mean age at enrollment was 18.2 years (58% were 16–18 years; 37% were 19–21 years, and 5% were 22–23 years at baseline). Fifty-six percent of the participants had a rheumatic condition (of these, 58% had inflammatory arthritis; 24% had lupus, Sjogren’s syndrome or mixed connective tissue disease; 12% had vasculitis and 6% had other conditions), An endocrinologic condition was the primary diagnosis in 21% (of these, 60% had diabetes, 10% had polycystic ovarian syndrome, 10% had pituitary dwarfism, and the remainder had Cushing’s syndrome, Klinefelter’s syndrome or hyperprolactinemia), A gastrointestinal disorder was present in and 23%, and all but one had inflammatory bowel disease. Ninety-six percent were unmarried and 82% reported living with parents. Just over half of the patients (53%) were not employed, and 81% were in school at time of enrollment. The majority of patients had private insurance supplied by their parents (65%).

**Table 2:** Baseline Characteristics of Cohort

	Overall (*N* = 89)	Transitioned (*N* = 39)	Non-transitioned (*N* = 44)	*P*-value
Age, mean (SD), year	18.3 (1.6)	18.8 (1.6)	18.0 (1.5)	0.03
Gender, No., %				0.54
Female	59 (66%)	14 (36%)	13 (30%)	
Male	30 (34%)	25 (64%)	31 (70%)	
Ethnicity, No., %				0.59
White	72 (81%)	31 (79%)	37 (84%)	
Non-white	17 (19%)	8 (21%)	7 (16%)	
Household income, No., %				0.43
< $25,000	11 (13%)	4 (10%)	7 (16%)	
$25,000–$45,999	16 (18%)	5 (13%)	9 (21%)	
$50,000–$74,999	13 (15%)	5 (13%)	8 (19%)	
$75,000–$99,999	6 (7%)	2 (5%)	4 (9%)	
$100,000–$150,000	8 (9%)	4 (10%)	4 (9%)	
> $150,000	7 (8%)	4 (10%)	1 (2%)	
Unknown	27 (31%)	15 (38%)	10 (23%)	
Type of insurance, No., %				0.44
Public	22 (26%)	8 (22%)	12 (29%)	
Private	62 (74%)	29 (78%)	29 (71%)	
Specialty, No., %				0.16
Rheumatology	50 (56%)	19 (49%)	28 (64%)	
Endocrinology	19 (21%)	8 (21%)	10 (23%)	
Gastroenterology	20 (23%)	12 (31%)	6 (14%)	
TRAQ domain 1 score, mean (SD)	3.1 (1.0)	3.2 (1.0)	3.0 (0.8)	0.37
TRAQ domain 2 score, mean (SD)	3.7 (0.7)	3.9 (0.5)	3.7 (0.9)	0.21
Duration of disease, mean (SD), year	4.8 (3.9)	5.2 (3.9)	4.5 (4.0)	0.27
Have discussed transition with provider, No., %	35 (41%)	21 (54%)	13 (33%)	0.06
Have seen provider independently, No., %				0.22
Always/Often/Sometimes	44 (51%)	23 (59%)	19 (45%)	
Never/Rarely	43 (49%)	16 (41%)	23 (55%)	

*Abbreviations*: *TRAQ* Transition Readiness Assessment Questionnaire

Only 40% of participants reported having discussed transition to adult care with their pediatric subspecialty provider. When asked if they saw their subspecialty provider independently for at least part of the visit, 33% reported never seeing the provider independently; 16%, rarely; 18%, sometimes; 25% often; and 7%, always.

### Baseline TRAQ scores

Baseline TRAQ domain 1 scores were not significantly related to age groups, gender, race, or disease specialty (all *P*-values >0.05.) TRAQ domain 2 scores were significantly increased in older age groups (*P*-value =0.02), but were not different based on gender, race, or disease specialty (all *P*-values >0.05).

### Transition follow up

Of the 89 participants, 39 transitioned to adult care, 44 did not transition, and transition status could not be determined for 6 participants. Baseline characteristics were similar between the transitioned and not-transitioned groups (Table 2). In univariate logistic regression analyses (Table 3), with each one-year increase in patient’s age, we noted an approximately 39% increase (OR 1.39, 95% CI 1.04–1.86; *P*-value: 0.028) in the odds of transition. Participants who had discussed transition with their provider at baseline had a 142% increase (OR 2.42; 95% CI: 0.97–6.04; *P*-value 0.058) in the odds of transition at the end of the study compared to those who did not have such a discussion. In the multivariable model with TRAQ Domain 2 (self –advocacy) score, age, and whether participant had discussed transition with the provider, none of the effects were significantly related to transition at the end of the study.

**Table 3:** Multivariable Analysis of Relationships between Baseline Characteristics and Transition

Predictor	Levels	Transition		OR	95% CI	*P*-value
		Yes	No			
TRAQ domain 1 score	One unit increase	3.22 ± 0.97	3.03 ± 0.83	1.28	0.79–2.10	0.32
TRAQ domain 2 score	One unit increase	3.90 ± 0.53	3.65 ± 0.86	1.67	0.85–3.27	0.14
Age (years)	One unit increase	18.77 ± 1.65	17.98 ± 1.49	1.39	1.04–1.86	0.03
Disease duration (years)	One unit increase	5.21 ± 3.85	4.50 ± 4.04	1.05	0.94–1.17	0.42
Gender	Female (*N* = 56)	25 (45%)	31 (55%)	0.75	0.30–1.88	0.54
	Male (*N* = 27)	14 (52%)	13 (48%)	1.0		
Race	White (*N* = 68)	31 (46%)	37 (54%)	0.73	0.24–2.25	0.59
	Non-white (*N* = 15)	8 (53%)	7 (47%)	1.0		
Insurance type	Private (*N* = 58)	29 (50%)	29 (50%)	1.5	0.53–4.21	0.44
	Public (*N* = 20)	8 (40%)	12 (60%)	1.0		
Specialty	Endocrinology (*N* = 18)	8 (44%)	10 (56%)	1.18	0.39–3.53	0.16
	Gastroenterology (*N* = 18)	12 (67%)	6 (33%)	2.95	0.94–9.22	
	Rheumatology (*N* = 47)	19 (40%)	28 (60%)	1.0		
Have seen provider independently	Sometimes/Often/Always (*N* = 42)	23 (55%)	19 (45%)	1.74	0.72–4.20	0.22
	Never/Rarely (*N* = 39)	16 (41%)	23 (59%)	1.0		
Have discussed transition with provider	Yes (*N* = 34)	21 (62%)	13 (38%)	2.42	0.97–6.04	0.06
	No (*N* = 45)	18 (40%)	27 (60%)	1.0		

*Abbreviations*: TRAQ Transition Readiness Assessment Questionnaire

**Fig. 1:**
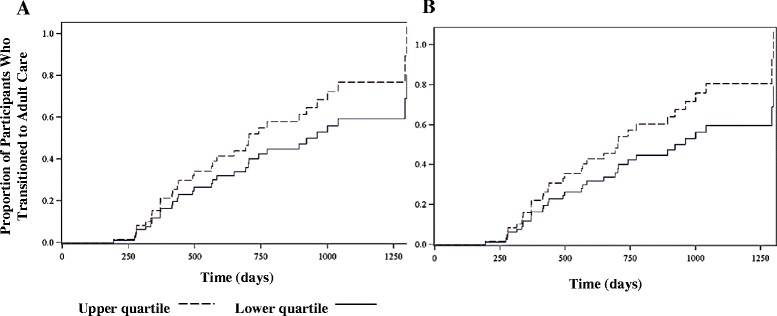
**a** shows the cumulative probability of transfer to adult subspecialist over the follow up period for the upper and lower quartiles of TRAQ 1 (self-management) Domain Score. **b** shows same results for TRAQ 2 Domain (self-advocacy) Score. Abbreviations: TRAQ = Transition Readiness Assessment Questionnaire

The time from study entry to the date of first visit with the adult provider was also analyzed in a multivariable Cox proportional hazards model with baseline TRAQ scores and age. (Fig. 1) The baseline TRAQ scores did not predict time to transition, though as noted in fig. 1, there was a possible trend showing sooner time to transition with higher TRAQ scores (all *P*-values >.05).

## Discussion

Because transition from pediatric to adult care is a vulnerable and risky time, there is significant impetus to improve transition processes and outcomes [2, 26]. The 2002 consensus guidelines proposed by the American Academy of Pediatrics, American College of Physicians and American Academy of Family Physicians-American Society of Internal Medicine called for transition to adult care to be a gradual process with a written, annually updated transition plan starting at age 14 [6, 15]. Subsequent guidelines published in 2011 recommend that transfer to adult provider occur between the ages of 18 and 21 [1].

As part of developing more reliable transition processes, these guidelines recommended that providers regularly assess transition readiness using an objective measure such as the TRAQ [15]. Assessment of transition readiness allows providers and patients to identify and nurture self-management abilities [27]. In addition to the TRAQ, other transition readiness tools include the UNC TRxANSITION scale [20], the AM I ON TRAC questionnaire [28], and the National Health Care Transition Center GotTransition assessment scale [29]. Some disease specific transition readiness assessment instruments have also been developed, including the Readiness for Adult Care in Rheumatology (RACER) [30],and the Diabetes Knowledge questionnaire [31].

This study reinforces existing data showing that adolescents and young adults are often not prepared for transition. In this cohort of 16 to 25 year olds, fewer than half reported ever having discussed transition to adult provider with their current subspecialty provider. Additionally, almost half (46%) either never or rarely saw the pediatric provider independently for part of the visit, although the AAP/AAFP/ACP guidelines recommend that adolescents begin seeing providers independently for at least part of the visit at age 14 [1].

To our knowledge, this is the first study to link transition readiness scores to transition outcomes. In our study, age was the only baseline variable that independently predicted transition over 3 years of follow up. In line with previous studies, TRAQ Domain 2 (self-advocacy) scores increased with age. However, in contrast with previous studies, self-advocacy scores did not differ significantly by gender [22]. The mean TRAQ Domain 1 (self-management) score was 3.1, reflecting the preparation stage of the Stages of Change Model [22]. The mean TRAQ Domain 2 (self-advocacy) score was higher at 3.9, moving toward the action stage. Neither baseline TRAQ domain score predicted transition or time to transition over 3 years of follow up; however there was a trend toward sooner time to transition amongst patients with highest quartile TRAQ scores. This study was limited to evaluating whether transition occurred, and did not address the quality of the transition process, associated cost or patient satisfaction. Validated tools which can provide useful and more comprehensive information about the quality of transition process are certainly needed but not currently available.

In this study, the TRAQ was only tested at baseline and results were not revealed to providers. However, the TRAQ was developed to be used over time with results influencing provider and patient collaboration in the development of self-management and self-advocacy skills. It is possible that change in TRAQ score over time would be more predictive of transition. Another possibility, first proposed by Mackie et al. is that the TRAQ has a “ceiling effect” in which it loses discriminative power in higher scoring individuals [32]. Because it had not yet been fully developed at the onset of this study, the updated TRAQ 5.0 instrument was not used [29].

While we attempted to test the TRAQ in a “real-world” clinical setting, we understand that our findings may not be generalizable. For example, our population was relatively small, largely white (81%) and non-Hispanic (91%). The age at transfer to adult care was not determined by hospital policy. Individuals up to age 25 years were allowed in the study which and therefore may include some older individuals than would typically be seen at other pediatric outpatient subspecialty clinics. However, 95% of the subjects in this study were 16 to 21 years of age at enrollment. Additionally it should be noted that TRAQ was ascertained only at a single visit with an adult specialty provider, and longitudinal TRAQ data may be more helpful in predicting when an individual will transition to adult care.

## Conclusion

Our results demonstrate that adolescents and young adults are often not prepared for transition according to recommended guidelines as only half of these 16–23 year olds reported seeing provider independently or discussing transition with provider. Validated transition assessment tools whose use clearly leads to improved outcomes are needed to promote successful transition processes. TRAQ scores did not significantly predict transition or time to transition in this study. More research is needed in larger cohorts to determine optimal methods to evaluate and improve preparedness for transition to adult care in adolescents and young adults with chronic diseases.

## Abbreviations

AAFP: American Association of Family Physicians
AAP: American Academy of Pediatrics
ACP: American College of Physicians
ARHQ: The Agency for Health Care Research and Quality
GI: gastroenterology
NS-CSHCN: National Survey of Children with Special Health Care Needs
RACER: Readiness for Adult Care in Rheumatology
TRAQ: Transition Readiness Assessment Questionnaire
